# Angiogenesis and Vasculogenesis in Health and Disease

**DOI:** 10.1155/2015/126582

**Published:** 2015-06-10

**Authors:** Alessio D'Alessio, Francesco Moccia, Jie-Hui Li, Alessandra Micera, Themis R. Kyriakides

**Affiliations:** ^1^Institute of Histology and Embryology, School of Medicine, Catholic University of the Sacred Heart, 00168 Rome, Italy; ^2^Department of Biology and Biotechnology “Lazzaro Spallanzani”, University of Pavia, 27100 Pavia, Italy; ^3^Department of Molecular and Cell Biology, Boston University Henry M. Goldman School of Dental Medicine, 650 Albany Street, Boston, MA 02118, USA; ^4^IRCCS-G. B. Bietti Foundation, 00198 Rome, Italy; ^5^Department of Pathology, Yale University School of Medicine, New Haven, CT 06510, USA

Angiogenesis is an important process that takes place during new blood vessel formation from preexisting ones. The first sign of vasculature occurs in the early phases of embryonic development when mesoderm-derived endothelial progenitor cells (EPCs) proliferate and form a primitive network of vessels during vasculogenesis. Vascular Endothelial Growth Factor (VEGF) is the most crucial factor in this process since it induces cell proliferation and migration. This primitive vascular structure, also known as the primary capillary plexus, is progressively remodeled in order to establish a mature circulatory network. Although vasculogenesis was thought to be restricted to embryonic development, recent studies support the existence of bone marrow-derived EPCs in adulthood that can contribute to neovascularization. In the adult, angiogenesis is mostly restricted to the ovarian cycle, injured tissues, and organ growth. Therefore, endothelial cells are considered stable and almost quiescent showing limited turnover in the adult vasculature. The lack of balance of pro- and antiangiogenic cues is considered one of the most crucial features that distinguish physiological from pathological angiogenesis. It is not surprising that we have witnessed an increase in interest in the field of angiogenesis research during the last decades with the main focus on trying to understand how to modulate blood vessel growth in order to develop new clinical approaches for a broad range of angiogenesis-related diseases. The main focus of this special issue is to highlight the complicated mechanism of angiogenesis in many different fields of cell biology. The special issue, which compiles a series of seven original research papers and two review articles, covers relevant aspects of vascular biology that are deeply discussed by experts in the field.

N. Irrera et al. investigated the effect of EPO-*α* and EPO-Z, two biosimilar recombinant human erythropoietins, on angiogenesis and cell proliferation in an experimental model of burn injury. Their study shows that although EPO-*α* and EPO-Z were both able to accelerate wound repair and angiogenesis, EPO-*α* was more effective in achieving complete skin regeneration. Authors hypothesize that the higher efficacy of EPO-*α* might be ascribed to an advantageous conformational structure, which renders this molecule more efficient in inducing cell proliferation and skin remodeling.

The interesting research article by I. Pafumi et al. describes a novel calcium-dependent machinery activated through the angiopoietin-1/2-tie receptor system. They employed the widely used human umbilical vein endothelial cells (HUVECs). The research team that has long-standing experience in the study of the intracellular calcium machinery in different experimental models suggests a novel Ca^2+^-dependent mechanism activated by angiopoietins that controls important angiogenic processes, including cell migration and the formation of capillary-like structures* in vitro*.

W. D. Ito and collaborators generated a specific monoclonal antibody, namely, CTA 157-2, against membrane preparations of growing collateral vessels. They show that CTA 157-2 specifically binds to the cell surface and activates the proteasome on vascular resident EPCs. In particular, they propose a functional role of the 20S proteasome activity on EPCs function and vascular growth by reducing the extent of collateral proliferation* in vivo*.

The article by L. Gackowska et al. is an interesting study that evaluates the different expression patterns of adiponectin receptors 1 and 2 (AdipoR1, AdipoR2) in children with primary hypertension versus healthy patients. The authors employed different experimental approaches, including flow cytometry, PCR, and ELISA, to show that neutrophil AdipoRs upregulation is associated with early stages of vascular injury, hypertension severity, and low serum levels of adiponectin. These findings suggest the involvement of the innate immune system in the development and maintenance of primary hypertension in children.

The research article by J. L. de Carvalho and coauthors proposes an innovative approach in order to generate functional endothelial cells, derived from Human Adipose-Derived Stromal Cells (hASCs), which can be employed for vascular grafts. By using an experimental model consisting of decellularized rat aorta with preserved basal membrane, they employed hASC derived ECs to promote reendothelialization of the aorta.

B. Lorusso et al. focus on lymphatic endothelial cells. Indeed, similar to the blood vascular system, the lymphatic system plays an important role in a variety of physiological and pathological conditions including inflammation, wound healing, and the formation of tumor metastasis. Their study proposes an innovative approach to obtain purified and functional human lymphatic endothelial cells isolated from human lung (HL-LECs) of healthy patients. Authors underline the potential application of HL-LECs for understanding the mechanisms regulating the biology of the lymphatic system.

X. Zhang et al. investigate the potential therapeutic contribution of Oncostatin M (OSM) to angiogenesis after myocardial infarction in a knock-out animal model deficient in the OSM-specific receptor *β* subunit. Their results indicate that OSM treatment preserved cardiac function, prevented cardiac hypertrophy, and stimulated angiogenesis via upregulation of VEGF and bFGF in infarct border zone of ischemic myocardium.

Two interesting review articles are also reported in this special issue. In their review article, A. Zimna and M. Kurpisz first review some general concepts of angiogenesis, hypoxia, and the importance of O_2_ homeostasis in the vascular network followed by a revision of recent studies concerning the contribution of the Hypoxia-Inducible Factor-1 (HIF-1) pathway and hypoxia in both physiological and pathological angiogenesis. Another review article by D. A. de Souza Junior and colleagues covers the current knowledge of how mast cell proteases, in particular chymases and tryptases, are implicated in tumor angiogenesis.

